# Effects of selenium biofortification on crop nutritional quality

**DOI:** 10.3389/fpls.2015.00280

**Published:** 2015-04-21

**Authors:** Mario Malagoli, Michela Schiavon, Stefano dall’Acqua, Elizabeth A. H. Pilon-Smits

**Affiliations:** ^1^Department of Agronomy, Food, Natural Resources, Animals and the Environment, University of Padova, Padova, Italy; ^2^Department of Pharmaceutical and Pharmacological Sciences, University of Padova, Padova, Italy; ^3^Department of Biology, Colorado State University, Fort Collins, CO, USA

**Keywords:** selenium, plant biofortification, food, nutritional quality, secondary metabolites

## Abstract

Selenium (Se) at very low doses has crucial functions in humans and animals. Since plants represent the main dietary source of this element, Se-containing crops may be used as a means to deliver Se to consumers (biofortification). Several strategies have been exploited to increase plant Se content. Selenium assimilation in plants affects both sulfur (S) and nitrogen (N) metabolic pathways, which is why recent research has also focused on the effect of Se fertilization on the production of S- and N- secondary metabolites with putative health benefits. In this review we discuss the function of Se in plant and human nutrition and the progress in the genetic engineering of Se metabolism to increase the levels and bioavailability of this element in food crops. Particular attention is paid to Se biofortification and the synthesis of compounds with beneficial effects on health.

## The Importance of Selenium to Human and Animal Health

Selenium is an essential trace element for humans and animals, and some organic forms like methyl-selenocysteine (MeSeCys) appear to be particularly effective sources of dietary Se. Selenium is incorporated as selenocysteine (SeCys) at the active site of a wide range of selenoproteins involved in major metabolic pathways, such as thyroid hormone metabolism, antioxidant defense and immune function (Rayman, 2012). Low intake of Se in the diet may cause a number of diseases, including heart diseases, hypothyroidism, reduced male fertility, weakened immune system and enhanced susceptibility to infections and cancer (Hatfield et al., 2014; Roman et al., 2014). Selenium deficiency is thought to affect 800 million people worldwide. In livestock, Se deficiency is also responsible for the white muscle disease, with clinical signs that include lesions in skeletal and/or heart muscle. Selenium supplementation of grazing livestock is mandatory in USA and Canada, because there is a marked seasonal and soil-dependent variation in their Se nutrition. For most of the world human and livestock population, vegetables are an important source of Se intake. Thus, increasing Se content in food crops offers an effective approach to reduce the Se deficiency problem in humans and animals.

## Selenium Transport and Assimilation in Plants

**FIGURE 1:**
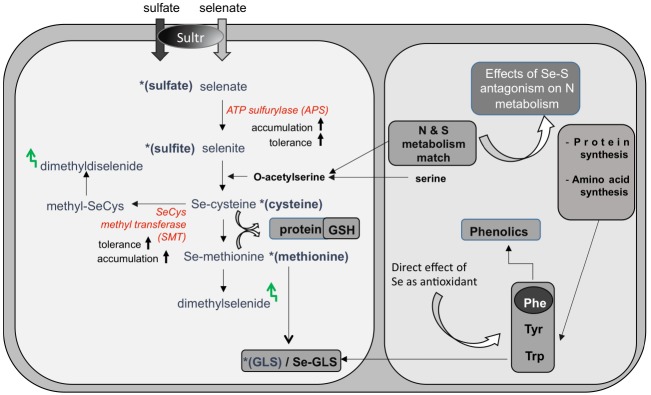
**Selenate (and sulfate) uptake and assimilation in plants.** Selenate is taken up by sulfate transporters (Sultr), and activated by ATP sulfurylase for further assimilation to selenocysteine (SeCys). SeCys can be further metabolized to selenomethionine and to volatile dimethylselenide. Non-hyperaccumulators often store selenate, because APS is a rate-limiting enzyme. Its overexpression resulted in enhanced Se accumulation and tolerance. Selenium hyperaccumulators methylate SeCys via the enzyme SeCys methyltransferase (SMT) and accumulate methyl-SeCys, a non-protein aminoacid. Methyl-SeCys may also be converted to volatile dimethyldiselenide. Expression of SMT in non-hyperaccumulators resulted in enhanced Se accumulation (as methylSeCys) and tolerance. Sulfur and nitrogen metabolic pathways interact at the level of -acetylserine. Changes in S assimilation induced by Se can in turn affect N metabolism, with respect to protein and amino acid synthesis. Amino acids methionine, phenylalanine (Phe), tyrosine (Tyr), and tryptophan (Trp) are precursors of glucosinolates (GLS) and Phe is a precursor for phenolics. Variation in the synthesis of these amino acids influence the production of nutraceutical compounds [glucosinolates (GLS) and phenolics]. In addition, Se can directly induce production of phenolics in plants.

While there is no proof of essentiality for Se in plants (Pilon-Smits et al., 2009), Se is readily taken up by plants in the form of selenate through the sulfate transporters (Figure 1). Due to their chemical similarities (Shibagaki et al., 2002; El Kassis et al., 2007), Se and sulfur (S) compete for the same transporters, and Se uptake is generally limited by high S levels. After uptake, selenate can access the sulfate assimilation pathway and be reduced via selenite to selenide (Figure 1). Selenide can be incorporated into the S-analog amino acid selenocysteine (SeCys), which may further be converted in three enzymatic steps to selenomethionine (SeMet; for a review, see Sors et al., 2005). The mistaken insertion of these Se-amino acids into proteins instead of cysteine and methionine may cause metabolic dysfunction (Sabbagh and Van Hoewyk, 2012). Incorporation of Se into proteins may be avoided by diverting Se to other, less toxic forms. Some plants accumulate the non-protein organic Se-compounds methylselenocysteine (MeSeCys), γ-glutamyl-MeSeCys and/or selenocystationine, sometimes to very high tissue levels without ill effects (Terry et al., 2000). Selenium can also be volatilized from plants in the forms of dimethylselenide or dimethyldiselenide, which are produced from SeMet and methyl-SeCys, respectively (Figure 1). The different selenocompounds found in plants have different toxicity levels and different nutritional value, with organic forms generally being more efficient in Se biofortification. Therefore, it is important to know which forms of Se are present in plant material used for nutritional supplementation. If we know which enzymes control the various metabolic steps it is also possible to genetically engineer more nutritious forms of Se in crop plants by enhancing the levels of critical enzymes.

## Selenium Biofortification Efforts

**FIGURE 2:**
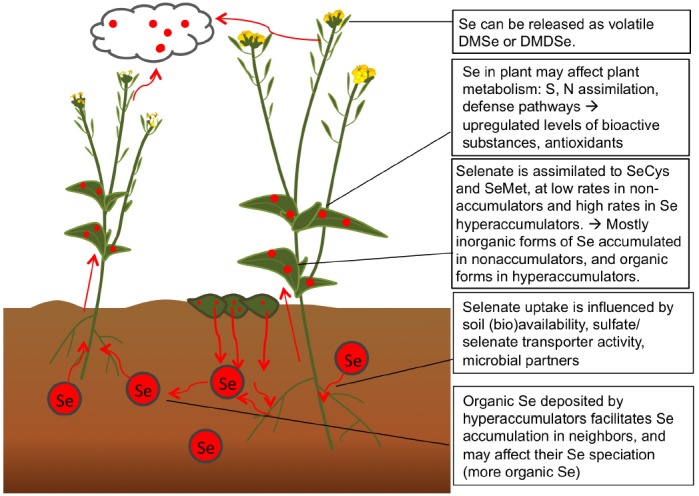
**Processes related to Se in the soil-plant system, relevant for Se biofortification.** Selenate is taken up from soil and assimilated (particularly by Se hyperaccumulators) to organic forms of Se. Some Se is accumulated and some volatiled as nontoxic dimethyl(di)selenide.

Selenium is chemically analogous to S and therefore accumulated by all plants to some extent, in all plant parts. The plant Se levels found in nature and in crops depends on soil Se abundance and the levels of competing S compounds (Figure 2). In addition, plant Se concentrations at a given seleniferous site, i.e., a site containing more than 1 (and up to 100) mg Se kg^–1^ soil, may vary over 100-fold between plant species (Galeas et al., 2007). Different plant species differ with respect to their capacity to accumulate Se, which likely correlates with their expression levels of sulfate transporters. Plant species also vary with respect to which forms of Se they accumulate due to the presence and activity of various S/Se metabolic enzymes. Selenium biofortification efforts may make use of this natural variation between plant species, and choose crop species that naturally tend to contain higher Se (and S) levels, such as *Brassica* and *Allium* species (Terry et al., 2000). Since Se biofortification is most effective when organic Se is supplied, plant species known to accumulate organic forms of Se may be preferred, including broccoli and garlic (Lyi et al., 2005; Hsu et al., 2011). Care has to be taken to not supply unnecessary S in Se-fortified crop production, since S will reduce Se uptake. In soils where Se levels are very low, as e.g., in Finland, the United Kingdom, parts of China, and New Zealand (Chen et al., 2002; Broadley et al., 2006; Alfthan et al., 2014), it is not enough to just plant Se-accumulating crop species, but also necessary to provide inorganic Se as fertilizer for the crop. This practice is in effect in Finland since the 1980s, and has led to significantly enhanced blood Se levels in the general population (Alfthan et al., 2014). Whether this is concomitant with positive health effects remains to be investigated; a complicating factor is that there is no reference population. In Se-deficient areas of China, too, Se biofortification of crops is practiced to prevent the devastating Keshan disease still prevalent in vast areas, which is characterized by cardiomyopathy caused by Se deficiency (Bañuelos et al., 2013).

## Genetic Engineering of Plant Se Metabolism and its Potential for Biofortification

Genetic engineering, which has been shown to enhance Se accumulation, tolerance, and volatilization by plants, has focused on S-related enzymes. First, overexpression in *Brassica juncea* of ATP sulfurylase (APS), a key enzyme for selenate-to-selenite transition, resulted in enhanced selenate reduction: the transgenic APS plants accumulated organic Se (likely methyl-SeCys) when supplied with selenate, while wildtype controls accumulated selenate (Pilon-Smits et al., 1999). The APS transgenics accumulated and tolerated more Se as well (Figure 1). In another approach, SeCys methyltransferase (SMT) was overexpressed in *A. thaliana* and *B. juncea* (Ellis et al., 2004; LeDuc et al., 2004). The SMT transgenics showed enhanced Se accumulation, and the form was methyl-SeCys (Figure 1). In both APS and SMT transgenics more Se is accumulated, and their form of Se is more suitable for biofortification (Figure 2).

When APS and SMT *B. juncea* transgenics were crossed to create double-transgenic plants, these accumulated up to 9 times higher Se levels than wild type (LeDuc et al., 2006). Most of the Se in the double transgenics was in the form of methyl-SeCys: the APSxSMT plants accumulated up to eightfold more methyl-SeCys than wild type and nearly twice as much as the SMT transgenics.

When grown on naturally seleniferous soil in a greenhouse pot experiment, the APS transgenics accumulated Se to threefold higher levels than wildtype *B. juncea* (Van Huysen et al., 2004). In two field experiments carried out on selenate-contaminated soil in central California, the APS transgenics accumulated fourfold higher Se levels than wildtype *B. juncea*, and SMT transgenics showed twofold higher Se levels (Bañuelos et al., 2005, 2007). Biomass production was comparable for the different plant types. Thus, genetic engineering has produced new genotypes of *B. juncea* with enhanced Se accumulation and higher levels of nutritious organic Se, all promising for use as Se-fortified foods.

In addition to the S assimilation enzymes, sulfate transporters may be potential targets of genetic engineering; selenate transporters from Se hyperaccumulators will be particularly interesting in this respect.

## Effects of Se Biofortification on Secondary Plant Compounds

Variations in plant S uptake and assimilation induced by Se may cause changes in the synthesis of S-secondary compounds with nutritional value, such as glucosinolates (GLS), which function in plant defense against insects and herbivores (Figure 1). The hydrolysis of GLS within cells produces isothiocyanates, which act as cancer-preventing agents in mammals (Dinkova-Kostova, 2013).

Because S nutrition is strictly associated with N metabolism, Se can exert an additional effect on the synthesis of proteins and amino acids, as well as on N-secondary compounds with free radical scavenging activities, like phenolics (Figure 1). Amino acids such as methionine, phenylalanine (Phe), tyrosine (Tyr) and tryptophan (Trp) are precursors of GLS. Furthermore, Phe is the substrate for phenolics biosynthesis. Variation in the synthesis of these amino acids influence the production of both types of beneficial compounds.

Several studies examined how Se enrichment of plants affects their content in these phytochemicals (Robbins et al., 2005; Barickman et al., 2013; Schiavon et al., 2013). Tomato (*Solanum lycopersicon* L.) plants and *Brassica* species in particular, contain high levels of phenolic compounds. Additionally, *Brassica* spp. are rich in glucosinolates (GLSs).

In broccoli (*Brassica oleracea* L.), Se fertilization was shown to reduce the amount of total phenolic acids, without altering the profile distribution of specific compounds (Robbins et al., 2005). In contrast, Se at low dosages (5 and 10 μM) increased the leaf phenolic content of hydroponically grown tomato plants (Schiavon et al., 2013). Furthermore, the supply of selenate via foliar spray at 2 and 20 mg Se plant^–1^ resulted in Se-biofortified tomato fruits, with enhanced levels of the antioxidant flavonoids naringenin, chalcone and kaempferol (Schiavon et al., 2013).

Selenium fertilization may also affect the levels of GLS, a class of secondary plant S compounds. GLS may have anticarcinogenic properties, based on studies using experimental *in vitro* and *in vivo* models, but can also cause toxicity at elevated levels (Assayed and Abd El-Aty, 2009). The presence of GLS and GLS-metabolites at high level in animal feed can cause the decrease in growth and production, affecting organs such as liver, kidney, lungs and inducing morphological and physiological changes of thyroid (Tripathi and Mishra, 2007). Robbins et al. (2005) reported a weak reduction of indole, aliphatic, total glucosinolates, and glucoraphanin after Se fertilization, and a strong fall of sulforaphane production. A Se-related decrease of these compounds in broccoli was also observed by Barickman et al. (2013), but high levels of GLSs could be maintained with Se concentration lower than 0.8 mg L^–1^ or by increasing S concentration in the medium. Exposing plants to low Se concentrations can promote S uptake and assimilation in some species, including *B. juncea* (Harris et al., 2014), thus potentially increasing the level of S-organic compounds. However, while upregulating S uptake and assimilation, Se treatment was also found to upregulate genes involved in GLSs breakdown in *A. thaliana* (Van Hoewyk et al., 2008).

Recently, Ávila et al. (2013, 2014) showed the reduction of GLSs in the florets of broccoli treated with selenate, whereas in the sprouts GLS levels were not affected. Moreover, sprouts contained nearly sixfold higher content of the potent anticancer glucoraphanin than florets. Se-enriched sprouts were expected to exhibit greater potential anticancer activity because of high accumulation of SeMCys with similar glucosinolate production. *Brassica* crops supplied with selenate were able to form selenoglucosinolates, with a methylselenoalkyl group that was likely derived from selenomethionine (Matich et al., 2012). Selenoglucosinolates accounted for 60% of the concentrations of their S analogs (Matich et al., 2012). The production of selenoglucosinolates following Se-fertilization has implications for human health, as the synthetic Se-containing isothiocyanates are reported to be more potent anticancer compounds than their S counterparts (Emmert et al., 2010). As mentioned, the Se-GLS and/or GLS content must be monitored when plants or their residues are used for human or animal consumption, to avoid potential toxicity effects.

## Future Prospects

Studies so far indicate that it is possible to maximize multiple bioactive components in a single plant. However, because in some cases the accumulation of Se may interfere with the production of some classes of phytochemicals, the Se biofortification programs must consider the interactions between Se and the main metabolic pathways of the plant. Particular attention should be paid to the reciprocal effects of Se and S on their accumulation and assimilation into organic compounds. In this context, managing S concentration during Se fertilization to vary S:Se ratios could be envisioned as a strategy to increase Se to beneficial dietary levels in plants without compromising GLS and other health-promoting compound contents.

An interesting new area of research involves the use of plant-microbe interactions to enhance Se biofortification. Another avenue to explore is the cultivation of Se fortified crops on seleniferous soil, thereby improving the amenity of that soil for further agriculture, and using the produced biomass to fortify the diets of people (and their livestock) in Se-deficient areas. Finally, since different plant species appear to be able to influence their neighboring plants’ Se accumulation and perhaps speciation (El Mehdawi et al., 2012), it will be interesting to further explore the potential of various co-cropping techniques to optimize crop Se biofortification and nutritional quality.

### Conflict of Interest Statement

The authors declare that the research was conducted in the absence of any commercial or financial relationships that could be construed as a potential conflict of interest.
